# Depression Impairs Level of Functioning in Chronic Kidney Disease Inpatients: A Case-Control Study

**DOI:** 10.7759/cureus.16017

**Published:** 2021-06-29

**Authors:** Anuj Virani, Rushi P Shah, Goher Haneef, Asma T Khan, Caroline C Dias, Kristal N Pereira, Siddharth Gupta, Prerna Sharma

**Affiliations:** 1 Family Medicine, Windsor University School of Medicine, Cayon, KNA; 2 Medicine, Byramjee Jeejeebhoy Medical College, Ahmedabad, Rajkot, IND; 3 Internal Medicine, University of Health Sciences, Lahore, PAK; 4 Emergency Medicine, University of Cincinnati Medical Center, Cincinnati, USA; 5 Internal Medicine, Larkin Community Hospital, South Miami, USA; 6 Psychiatry, Yenepoya Medical College and Hospital, Toronto, CAN; 7 Internal Medicine, Terna Medical College, Mumbai, IND; 8 Internal Medicine, Sri Guru Ram Das Institute of Medical Sciences and Research, Amritsar, IND; 9 Psychiatry, Government Medical College, Amritsar, IND

**Keywords:** severe mdd, disease mortality, chronic kidney disease (ckd), depression, hospital outcomes

## Abstract

Objectives

To evaluate the difference in demographics and clinical correlates during hospitalization for chronic kidney disease (CKD) between patients with depression and those without depression, and its impact on the severity of illness and in-hospital mortality.

Methods

We conducted a case-control study and included 2,296 adult inpatients (age ≥18 years) with a primary discharge diagnosis of CKD using the nationwide inpatient sample (NIS). We used propensity score matching to extract the cases i.e., CKD inpatients with depression (N = 1,264) and the controls i.e. CKD inpatients without depression (N = 1,032). The matching was done based on demographic characteristics of age at admission, sex, race, and median household income. Our outcomes of interest are the severity of illness and all-cause in-hospital mortality. All patient refined drg (APR-DRG) are allocated using health information systems software by the NIS and the severity of illness within each base APR-DRG was classified into minor, moderate, or major loss of body functions. Binomial logistic regression analysis was conducted to find the odds ratio (OR) of association for major loss of function in CKD inpatients with depression, and this model was adjusted for potential confounders of congestive heart failure (CHF), coronary artery disease (CAD), diabetes, hypertension, obesity, and tobacco abuse, and utilization of hemodialysis.

Results

A higher proportion of CKD inpatients with depression had a statistically significant higher prevalence of major loss of function (49.8% vs. 40.3% in non-depressed). There was a statistically significant difference with higher utilization of hemodialysis in CKD inpatients with depression (76.2% vs. 70.7% in non-depressed). The all-cause in-hospital mortality rate was lower in CKD inpatients with depression (2.1% vs. 3.5% in non-depressed). After controlling the logistic regression model for potential comorbidities and utilization of hemodialysis, depression was associated with increased odds (OR 1.46; 95% CI 1.227 - 1.734) for major loss of function versus in non-depressed CKD inpatients

Conclusion

Comorbid depression increases the likelihood of major loss of functioning in CKD inpatients by 46%. Treating depression can allow patients to better cope emotionally and physically with CKD and other comorbidities and significantly improve the patient’s quality of life (QoL) and health outcome.

## Introduction

Chronic kidney disease (CKD) has risen to become one of the most common ailments within the United States (US) population. Currently, one in seven adults has CKD, which is 15% of the population and estimated to be about 37 million individuals. It is more common in people aged above 65 years (38%) than the age group of 45-64 (12%), or 18-44 (6%), and is more common in females [1]. According to the world health organization (WHO) bulletin, around 2.6 million people received dialysis in 2010, and the current trends indicate that this number will nearly double by 2030. This ascension will not only affect the well-being of many lives but prove to be costly as well. In 2015, Medicare spent 64 billion dollars for the treatment of CKD and 34 billion dollars for end-stage renal disease (ESRD). It is also found that CKD is the major contributor to mortality and morbidity due to underlying comorbidities like diabetes mellitus and hypertension [2].

Factors associated with CKD, such as increased oxidative stress, uremic toxins, anemia, and hyperparathyroidism can play a role in the overall genitive decline in patients. One in five adults with CKD in the US suffers from depression. Patients on dialysis have a higher prevalence of depression and a higher risk of hospitalizations due to psychiatric dysfunction compared to their pre-dialysis and post-transplant counterparts [3]. Behavioral factors such as the burden of self-care decreased day-to-day functionality, and the psychological impact of having a chronic illness also contribute to developing depression [4]. The prevalence of major depressive episodes in CKD patients is about 20% which is greater than those reported for patients with other chronic diseases including diabetes (11%), congestive heart failure (CHF, 14%), and coronary artery disease (CAD, 16%) [5]. 

Increased hospitalizations and the burden of chronic illness accompanied by depression lead to a poor quality of life (QoL), increased mortality rate, and decline in overall functioning among CKD patients. About half of adults with CKD have comorbid depression and they have a 9% higher likelihood of receiving antidepressant treatment [6]. It is important to screen and correctly diagnose depression early in the course of the disease to comprehensively improve the health outcome among CKD patients. We conducted a case-control study to evaluate the impact of depression on the hospitalization outcomes of the severity of illness and in-hospital mortality in CKD patients.

## Materials and methods

Study sample

Our initial study sample included 62,130 adult inpatients (age 18 years and above) with a primary discharge diagnosis of CKD using the nationwide inpatient sample (NIS). The NIS is the largest inpatient database and represents a sample of 4,411 non-federal community hospitals across 44 states in the United States [7]. 

CKD inpatients with a comorbid diagnosis of depression were identified using the international classification of diseases, clinical modification (ICD-9-CM) diagnostic codes of 296.20-296.26 or 296.30-296.36. The prevalence of depression in CKD inpatients was 10.17% (6,320 out of 62,130). We used propensity score matching to extract the cases i.e., CKD inpatients with depression (N = 1,264) and the controls i.e. CKD inpatients without depression (N = 1,032). The matching was done based on demographic characteristics of age at admission, sex, race, and median household income with a match tolerance specify value of zero for exact matches.

Variables

The demographic variables included in this study were age at admission, sex, race, and median household income. Comorbidities are considered as co-diagnoses in the patient records and we included CHF, CAD, diabetes (with chronic complications and uncomplicated), hypertension, obesity, and tobacco abuse. The hospitalization outcomes of interest included are the severity of illness, and all-cause in-hospital mortality, and utilization of hemodialysis. All patient refined drg (APR-DRG) are allocated using health information systems software by the NIS and the severity of illness within each base APR-DRG was classified into minor, moderate, or major loss of body functions [8].

Statistical analysis

We compared the distributions of demographic characteristics, comorbidities, and hospitalization outcomes in CKD inpatients without versus with depression by performing Pearson’s chi-square test. Next, we measured the differences in continuous variables i.e., age and number of chronic conditions in CKD inpatients without versus with depression using the independent sample t-test. Then, binomial logistic regression analysis was conducted with nagelkerke r square of 0.107 to find the odds ratio (OR) of association for major loss of function in CKD inpatients with depression, and this model was adjusted for potential confounders of CHF, CAD, diabetes (with chronic complications and uncomplicated), hypertension, obesity, and tobacco abuse, and utilization of hemodialysis. All analyses were conducted using the Statistical Package for the Social Sciences (SPSS) version 26.0 (IBM Corp., Armonk, NY) and statistical significance was set at a two-sided P value <0.05.

Ethical approval

The NIS is publicly available de-identified data with the protection of patients, physicians, and hospital-related information; henceforward, we were not required to take institution review board permission for our study [7].

## Results

The majority of the CKD inpatients with depression were females (57.4%) and were prevalent amongst Whites (49.0%), followed by Hispanics (26.1%) and Blacks (19.4%), and low-income families with median household income below the 50th percentile. 

CKD inpatients with depression had a higher mean number of comorbidities (9.4 vs. 7.1 in non-depressed). Though there existed a statistically non-significant difference in the comorbidities (CHF, CAD, diabetes, hypertension, obesity, and tobacco abuse) between CKD inpatients with depression versus without depression.

A higher proportion of CKD inpatients with depression had a statistically significant higher prevalence of major loss of function (49.8% vs. 40.3% in non-depressed). There was a statistically significant difference with higher utilization of hemodialysis in CKD inpatients with depression (76.2% vs. 70.7% in non-depressed). The all-cause in-hospital mortality rate was lower in CKD inpatients with depression (2.1% vs. 3.5% in non-depressed) as shown in Table 1.

**Table 1:** Differences in chronic kidney disease inpatients by depression N: number of inpatients; SD: standard deviation

Variable	Depression (no)	Depression (yes)	P value
N	1032	1264	-
Mean age (SD), in years	57.0 (15.3)	55.9 (15.9)	0.094
Sex, in %			
Male	43.6	42.6	0.616
Female	56.4	57.4	
Race, in %			
White	54.6	49.0	0.348
Black	19.0	19.4	
Hispanic	22.4	26.1	
Others	4.1	5.4	
Median household income, in %			
0 – 25^th^ percentile	40.0	37.0	0.423
26^th^ – 50^th^ percentile	27.6	30.3	
51^st^ – 75^th^ percentile	20.6	20.4	
76^th^ – 100^th^ percentile	11.7	12.3	
Mean number of chronic conditions (SD)	7.1 (3.1)	9.4 (3.0)	<0.001
Comorbidities, in %			
Congestive heart failure	25.2	26.7	0.425
Coronary artery disease	27.2	27.1	0.927
Diabetes, uncomplicated	22.8	23.4	0.715
Diabetes with chronic complications	24.5	24.0	0.762
Hypertension	4.8	4.5	0.704
Obesity	12.1	13.4	0.370
Tobacco abuse/dependence	7.2	8.6	0.201
Severity of illness, in %			
Minor loss of function	18.2	15.3	<0.001
Moderate loss of function	41.5	34.9	
Major loss of function	40.3	49.8	
Utilization of hemodialysis	70.7	76.2	0.003
In-hospital mortality, in %	3.5	2.1	0.035

Comorbidities were associated with a significant increase in the odds for major loss of function. Among medical comorbidities, increased risk of major loss of function was seen in CHF (OR 2.29; 95% CI 1.872 - 2.805) followed by diabetes, uncomplicated (OR 1.67; 95% CI 1.341 - 2.068) and diabetes with chronic complications (OR 1.47; 95% CI 1.183 - 1.820). Comorbid hypertension, obesity, and tobacco abuse/dependence was not a significant risk factor for major loss of functioning in CKD inpatients. After controlling the logistic regression model for potential comorbidities and utilization of hemodialysis, depression was associated with increased odds (OR 1.46; 95% CI 1.227 - 1.734) for major loss of function versus in non-depressed CKD inpatients as shown in Table 2.

**Table 2:** Logistic regression analysis for major loss of function

Comorbidities	Odds ratio	95% Confidence interval	P value
Depression	1.46	1.227 – 1.734	<0.001
Congestive heart failure	2.29	1.872 – 2.805	<0.001
Coronary artery disease	1.42	1.166 – 1.735	<0.001
Diabetes, uncomplicated	1.67	1.341 – 2.068	<0.001
Diabetes with chronic complications	1.47	1.183 – 1.820	<0.001
Hypertension	1.17	0.768 – 1.779	0.468
Obesity	1.25	0.966 – 1.629	0.089
Tobacco abuse/dependence	1.17	0.857 – 1.609	0.319
Utilization of hemodialysis	1.42	1.160 – 1.741	0.001

## Discussion

Patients with chronic illness have a poor QoL and a higher likelihood of psychiatric illnesses and experience depressive and anxiety symptoms [9,10]. Some factors that lead to a worsening of depression include long-term medication treatments, frequent hospitalizations, and the burden of handling chronic illness. Depression in CKD patients undergoing dialysis is related to biological, psychological, and social factors [11]. The increased cytokine levels and alteration of neurotransmitters by uremia, and comorbid vitamin b12 deficiency and anemia, and genetic predisposition are some known biological factors [12-14]. Based on animal and human studies, urea accumulation in the brain is an independent factor causing depression, bypassing psychosocial stress [13].

CKD inpatients with depression had a higher mean number of chronic conditions [7,8]. Depression was not associated with an increased in-hospital mortality rate compared to the non-depressed group in our study. Drayer et al. reported the association of depression with mortality in a cohort of 62 chronic hemodialysis patients. About two-thirds of the patients categorized as "depressed" did not meet the criteria for major depressive disorder and experienced “minor depression.” Selection bias may have further confounded this study considering that recruitment was not done consecutively and resulted in the inclusion of morbidly ill and challenging CKD patients who would have a greater propensity for poor outcomes [15]. 

Depression has been found to increase the risk of de novo CAD. In addition, there is also an increased risk of cardiovascular morbidity and mortality in those with pre-existing CAD [16,17]. Based on animal models and human studies, various potential mechanisms for this correlation have been posited, including behavioral factors. These aforementioned factors range from treatment adherence to lifestyle choices, such as smoking, poor nutrition, and inactivity. As a result, physiological factors play a role, such as changes in platelet reactivity, clotting activation, autonomic nervous system dysregulation, and hypothalamic pituitary adrenal axis, endothelial dysfunction, and alterations in immune response and inflammation [16-18]. In our study, we did not find any statistically significant difference in the prevalence rate of CAD, CHF, diabetes, hypertension, and obesity between CKD patients with versus without depression.

About half of the CKD inpatients with depression in our study had a major loss of body functioning and were at 1.5 times increased odds for the greater severity of the illness compared to the non-depressed patients [19,20]. Depression among CKD patients causes a decrease in QoL and loss of day-to-day functionality, which coupled with comorbidities can lead to a rapid decline in physical health and adverse outcome [17,19,20]. Depressed patients have more hopelessness and are at a higher likelihood of experiencing sarcopenia within one year of onset of depressive symptoms. Due to sarcopenia, these patients have severe loss of muscle mass leading to severe difficulties in performing tasks and impaired functioning [21].

Other factors that can worsen depressive symptoms in CKD patients include somatic symptoms and lack of ability to cope with the distress of dealing with chronic progressive disease, and poor social and emotional support. A study conducted on 227 patients on hemodialysis in northern China revealed that living alone, lack of social/family support, and lack of formal education led to the worsening of depressive symptoms. CKD patients on hemodialysis deal with unpredictable exacerbation of their symptoms, hospitalizations, and dialysis visits. These patients visualize their situation with feelings of hopelessness and use acceptance and resignation as a coping style due to which they were more vulnerable to worsening depression [22]. Depression with CKD leads to dysregulation of the hypothalamic-pituitary axis activity, resulting in increased cortisol and norepinephrine excretion which can affect patient’s nutritional health, worsen inflammation process, and further exacerbate atherosclerosis in patients undergoing dialysis [23-26]. CKD patients with depression have derogatory behavior which can lead to exacerbation of comorbid conditions (hypertension, diabetes, and hypercholesterolemia), and nonadherence to dialysis and prescribed medications. This causes the accumulation of toxins in the blood leading to adverse outcomes including increased mortality and poor prognosis [27-29].

Our study should be considered with some limitations. First, as it was a case-control and retrospective study it cannot establish a cause-effect or temporal relationship between CKD and depression. Second, all information of this study is collected from an administrative database, so patient-related clinical information of the stage and treatment of CKD, and severity and treatment of depression is unavailable. Also, due to the use of diagnostics codes, there may be under-reporting of comorbidities.

## Conclusions

Comorbid depression increases the likelihood of major loss of functioning in CKD inpatients by 46%. These at-risk patients also have higher utilization of hemodialysis compared to the CKD patients without depression. Physicians should vigilantly screen CKD patients for depression for early diagnosis and management to allow patients to better cope emotionally and physically with CKD and other comorbidities and improve the patient’s QoL and health outcome.
